# Genomic Profiling of Uterine Aspirates and cfDNA as an Integrative Liquid Biopsy Strategy in Endometrial Cancer

**DOI:** 10.3390/jcm9020585

**Published:** 2020-02-21

**Authors:** Carlos Casas-Arozamena, Eva Díaz, Cristian Pablo Moiola, Lorena Alonso-Alconada, Alba Ferreiros, Alicia Abalo, Carlos López Gil, Sara S. Oltra, Javier de Santiago, Silvia Cabrera, Victoria Sampayo, Marta Bouso, Efigenia Arias, Juan Cueva, Eva Colas, Ana Vilar, Antonio Gil-Moreno, Miguel Abal, Gema Moreno-Bueno, Laura Muinelo-Romay

**Affiliations:** 1 Translational Medical Oncology Group (Oncomet), Health Research Institute of Santiago de Compostela (IDIS), University Hospital of Santiago de Compostela (SERGAS), Trav. Choupana s/n, 15706 Santiago de Compostela, Spain; carlos.casas_95@hotmail.es (C.C.-A.); Alicia.Abalo.Pineiro@sergas.es (A.A.); jfcueva@gmail.com (J.C.); miguel.abal.posada@sergas.es (M.A.); 2 Foundation MD Anderson International, C/Gómez Hemans 2, 28033 Madrid, Spain; eva.diaz@mdanderson.es (E.D.); saraoltra4@gmail.com (S.S.O.); gmoreno@iib.uam.es (G.M.-B.); 3 Biomedical Research Group in Gynecology, Vall d’Hebron Research Institute (VHIR), Universitat Autonoma de Barcelona, 119-129 Pg. Vall d’Hebron, 08035 Barcelona, Spain; cristian.pablo@vhir.org (C.P.M.); scabrera.vhebron@gmail.com (S.C.); eva.colas@vhir.org (E.C.); antonioimma@yahoo.com (A.G.-M.); 4 Nasasbiotech, S.L., Canton Grande 3, 15003 A Coruña, Spain; lorena.alonso@nasasbiotech.com (L.A.-A.); alba.ferreiros@nasasbiotech.com (A.F.); 5 Department of Gynecology, MD Anderson Cancer Center, 28029 Madrid, Spain; jsantiagog@hotmail.es; 6 Department of Gynecology, University Hospital of Santiago de Compostela (SERGAS), Trav. Choupana s/n, 15706 Santiago de Compostela, Spain; vitosampayo@hotmail.com (V.S.); efi.arias@yahoo.com (E.A.); ana.vilar@telefonica.net (A.V.); 7 Department of Pathology, University Hospital of Santiago de Compostela (SERGAS), Trav. Choupana s/n, 15706 Santiago de Compostela, Spain; marta.bouso.montero@sergas.es; 8 Centro de Investigación Biomédica en Red de Cáncer (CIBERONC), Monforte de Lemos 3-5, 28029 Madrid, Spain; 9 Department of Biochemistry, Autonomic University of Madrid (UAM), Biomedical research Institute ‘Alberto Sols’ (CSIC-UAM), IdiPaz, Arzobispo Morcillo 4, 28029 Madrid, Spain

**Keywords:** endometrial cancer, uterine aspirates, circulating biomarkers, circulating tumor DNA (ctDNA), circulating tumor cells (CTCs), PDX models, targeted therapies

## Abstract

The incidence and mortality of endometrial cancer (EC) have risen in recent years, hence more precise management is needed. Therefore, we combined different types of liquid biopsies to better characterize the genetic landscape of EC in a non-invasive and dynamic manner. Uterine aspirates (UAs) from 60 patients with EC were obtained during surgery and analyzed by next-generation sequencing (NGS). Blood samples, collected at surgery, were used for cell-free DNA (cfDNA) and circulating tumor cell (CTC) analyses. Finally, personalized therapies were tested in patient-derived xenografts (PDXs) generated from the UAs. NGS analyses revealed the presence of genetic alterations in 93% of the tumors. Circulating tumor DNA (ctDNA) was present in 41.2% of cases, mainly in patients with high-risk tumors, thus indicating a clear association with a more aggressive disease. Accordingly, the results obtained during the post-surgery follow-up indicated the presence of ctDNA in three patients with progressive disease. Moreover, 38.9% of patients were positive for CTCs at surgery. Finally, the efficacy of targeted therapies based on the UA-specific mutational landscape was demonstrated in PDX models. Our study indicates the potential clinical applicability of a personalized strategy based on a combination of different liquid biopsies to characterize and monitor tumor evolution, and to identify targeted therapies.

## 1. Introduction

In the developed world, endometrial cancer (EC) is the most common neoplasm of the female genital tract, and its incidence is increasing [1]. Although the prognosis of EC is generally good, many cases are diagnosed at advanced stages; these cases are usually high-grade carcinomas that are ultimately more likely to recur and are associated with high mortality. In addition, a non-negligible 2.5%–3% of low-risk patients with EC have recurrent disease [2]. Overall, the prognosis for recurrent endometrial cancer is poor, especially for the 50% of advanced ECs with extra-pelvic disease recurrence [3]. Much effort has been devoted to generate a consensus on EC risk classification to promote consistency for future clinical trial design, including EC molecular characterization and integrating it into clinicopathologic profiling to develop prognostic and predictive biomarkers [4].

In parallel, the oncologic management of advanced disease has been revolutionized by the advent of liquid biopsy, i.e., the analysis of tumoral material shed from primary tumors and their metastatic sites into peripheral blood, such as circulating tumor cells (CTCs) and circulating tumor DNA (ctDNA). Major advantages of liquid biopsy analysis include its minimal invasiveness and ability to provide real-time information about the disease [5]. To date, the utility of liquid biopsy in EC has been scarcely explored [6]. The presence of CTCs has been shown to be limited in EC, and a small number of high-risk patients, mainly those with non-endometrioid high grade carcinomas, have been identified with Epithelial Cell Adhesion Molecule (EpCAM) positive CTCs in circulation at the time of diagnosis. Those studies—such as the one conducted by the European Network for Individualized Treatment in EC (ENITEC) Consortium, which found 22% CTC positivity in 32 high-risk patients with EC [7]—have been performed in limited cohorts. Circulating free DNA (cfDNA) and ctDNA have been described to be promising in EC management. In fact, elevated cfDNA levels have been detected in patients with EC [8]. Furthermore, targeted sequencing of commonly mutated genes in EC—such as *CTNNB1*, *KRAS*, *PTEN*, or *PIK3CA*—has also been performed on cfDNA at the time of surgery, thus leading to the identification of at least one mutation in more than 33% of patients with endometrioid-type endometrial carcinoma (EEC) [9]. In addition to peripheral blood—the prototypical form of liquid biopsy and the main source of clinically useful tumor material—uterine aspirates (UAs) are a minimally invasive alternative form of liquid biopsy with high relevance in gynecologic malignancies. Notably, UA can be used to identify the tumor mutational landscape and importantly, this genetic analysis also captures the high intratumor heterogeneity associated with EC [10]. In this regard, the recently developed PapSEEK test, applied to UA sampling, has shown that 81% of patients with EC have detectable mutations [11].

**Figure 1:**
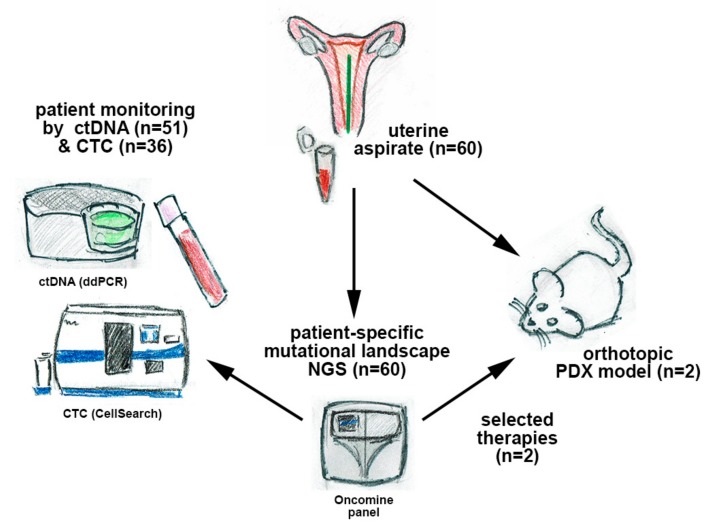
Scheme representing the workflow of the study with the samples and analyses performed.

In this work, we developed an integrated strategy using different types of liquid biopsy in patients with EC to provide valuable and complementary information for the management of advanced disease. For this purpose, we performed targeted next-generation sequencing (NGS) on UA samples from a cohort of 60 patients with EC and monitored patient-specific mutations by droplet digital PCR (ddPCR) in cfDNA. In addition, we evaluated the clinical utility of cfDNA and CTCs in these patients; designed targeted therapies based on the molecular landscape; and finally, validated the therapies in patient-derived xenografts (PDXs) generated after orthotopic implantation of UA (scheme in Figure 1).

## 2. Experimental Section

### 2.1. Patient Inclusion and Sample Collection

A total of 60 patients were recruited between January 2018 and July 2019 at the Gynecology Department of Vall d’Hebron University Hospital (Barcelona, Spain), the MD Anderson Cancer Center (Madrid, Spain), and the University Hospital of Santiago de Compostela (Santiago de Compostela, Spain). The EC cohort included low- to high-risk, grade 1–3, and stage I–IV cases, at first diagnosis or recurrence (Table 1). The study was carried out according to the rules of the Declaration of Helsinki of 1975, revised in 2013, and according to the standards for good clinical practice and other local ethical laws and regulations. Informed consent forms, approved by the pertinent ethical committees, were signed by all patients (Autonomic Galician Ethical Committee Code 2017/430, approval number PRAMI276–2018 of the Vall d’Hebron Ethical Committee).

UAs were obtained at surgery with a Cornier cannula and kept on ice until they were processed, always within 1 hour. Each UA was then homogenized with phosphate-buffered saline (PBS) at a 1:1 ratio and centrifuged at 4 °C for 20 min at 2500 × *g*. The supernatant and pellet were stored at −80 °C until use. Peripheral blood samples were also collected at surgery into CellSave Preservative tubes (Silicon Biosystems Inc, Huntington Valley, USA). A two-step centrifugation was performed to isolate the plasma. First, blood samples were centrifuged for 10 min at 1600 × *g* at room temperature. Supernatant was collected, avoiding the buffy coat, and then centrifuged again for 15 min at room temperature and 6000 × *g* to remove remaining cells. Plasma supernatants were stored at −80 °C until use.

The fraction containing the mononuclear cells obtained after the first centrifugation was used for CTC isolation with the CellSearch system (Menarini, Sylicon Biosystems, Bologna, Italy). This system allows for the isolation and enumeration of EpCAM-positive CTCs. After CTC isolation with the CellSearch Epithelial Circulating Tumor Cell Kit (Menarini, Silicon Biosystems Inc), cells were labeled with phycoerythrin-conjugated anti-cytokeratin (CK) antibodies, allophycocyanin-conjugated anti-CD45 antibodies, and 4,6-diamino-2-phenylindole (DAPI) to stain the nuclei (Figure S1). The CellTracks Analyzer (Menarini, Silicon Biosystems, Bologna, Italy) was used to acquire digital images of the three different fluorescent dyes with a 12-bit camera; the images were reviewed by trained operators to determine the CTC count.

### 2.2. DNA Extraction

DNA extraction from cells present in the pellet, obtained after UA processing, was performed with the MagMAX^TM^ Total Nucleic Acid Isolation Kit (Applied Biosystems, Foster City, California, USA), according to the manufacturer’s specifications. DNA from plasma samples was extracted with the QIAamp DNA Circulating Nucleic Acid Kit (Qiagen, Venlo, Netherlands), according to the manufacturer’s instructions. DNA samples were stored at −20 °C until use. The quantification of DNA from all samples was performed with the Qubit Fluorometer (Thermo Fisher Scientific, Waltham, MA, USA) and the Qubit DNA High-sensitivity Assay (Thermo Fisher Scientific, Waltham, MA, USA). Agilent’s TapeStation 2200 (Agilent Technologies, Santa Clara, CA, USA) was used to assess the fragment distribution of the extracted DNA (Figure S2A).

### 2.3. Targeted Sequencing of UA, Personalized Therapy Selection, and ddPCR Assays

Targeted sequencing of UA was performed with the Oncomine Comprehensive Panel v3 (Thermo Fisher, Pleasanton, CA, USA), and personalized therapies identified through an in silico study using various compound databases are detailed below.

To prepare amplicon libraries, we performed targeted sequencing of uterine multiplex PCR with the Ion AmpliSeq Library Kit 2.0 and Oncomine Comprehensive Panel v3 (Thermo Fisher, Pleasanton, CA, USA). For PCR, a total of 17 and 20 cycles were performed. The PCR template preparation and enrichment were performed with the Ion PGM Template OT2 200 Kit and Ion OneTouch 2 System. Finally, the Ion PGM Sequencing 200 Kit v2 and Ion PGM System (Life Technologies, Santa Clara, CA, USA) were used for DNA sequencing, according to the manufacturer’s protocols. Duplicates were analyzed for 10% of the samples and found to yield equivalent results.

For the bioinformatics analysis, alignment to the Hg19 human reference genome and variant calling were performed with Torrent Suite™ Software v.4.2.1 (Life Technologies, Santa Clara, CA, USA). All samples were sequenced and analyzed in comparable conditions. The mean coverage per sequenced sample was approximately 1500 reads per base. Variants with a Phred quality score field value less than 100 were considered as low-quality variants. The prediction of genomic variant effects on protein function was performed with the PROVEAN Genome Variants tool (http://provean.jcvi.org/index.php). Variants with possibly damaging or deleterious consequences, as predicted by at least one of the PROVEAN predictors, were considered to be of interest and were visually checked with Integrative Genomics Viewer (IGV) v.2.3.40, Broad Institute. Variants with a global minor allele frequency above 0.05 were considered as single nucleotide polymorphisms and were rejected (data from dbSNP, http://www.ncbi.nlm.nih.gov/SNP/).

### 2.4. Personalized Therapy Selection

To identify potential effective drugs on the basis of the mutational profile obtained from the targeted sequencing analysis, we performed an in silico study using the CTD (http://ctdbase.org/) and STITCH (http://stitch.embl.de/) compound databases. These databases contain peer-reviewed information about the effect of particular drugs on specific genes or signaling pathways, thus indicating the relationship between chemicals and proteins in the human context. In this analysis, we included the mutational status of each identified gene per case. The identified drugs were then used to treat PDX models, and standard protocol treatment was included as a control.

### 2.5. Detection of ctDNA with ddPCR

For each patient, specific ddPCR assays were designed and run on a QX-200 dPCR system (Bio-Rad, California, USA) using TaqMan chemistry, with primers and probes at final concentrations of 900 nM and 250 nM, respectively. For each patient-specific assay, one non-target control, one wild-type control (fragmented genomic DNA from a healthy donor), and one positive control (patient UA DNA) were used. Custom TaqMan assays were used (Bio-Rad, CA, USA) according to the variants discovered from tumor tissue sequencing. The linearity and the lower limit of detection (LOD) for all ddPCR assays used in the study were tested with dilutions of synthetic targeted sequences in a background of 20,000 copies of wild type (WT) DNA (mutant copies ranging from 0 to 40). All assays were run in triplicates and to assure their performance, the Pearson correlation between the estimated and experimental mutant copies detected was calculated (Pearson r = 0.995) (Figure S2B–C).

PCR was performed with the ddPCR Supermix for probes (Bio-Rad, Santa Rosa, CA, USA) and partitioned into a median of 50,000 droplets per sample (run in triplicates) in an automated droplet generator (Bio-Rad, CA, USA), according to the manufacturer’s instructions. Emulsified PCR reactions were run on 96-well plates on a C1000 Touch^TM^ thermal cycler (Bio-Rad, CA, USA) by incubating the plates at 95 °C for 10 min followed by 40 cycles of 95 °C for 15 sec; the specific assay extension temperature for 60 sec; and 98 °C for 10 min. The temperature ramp increment was 2.5 °C/sec for all steps. Plates were read on a Bio-Rad QX-200 droplet reader with Bio-Rad’s QuantaSoft v1.7.4 software to quantify the number of droplets positive for mutant DNA, wild-type DNA, both, or neither. A minimum of 30,000 positive droplets across wells were required for a valid assay, and a minimum of five, single FAM-positive droplets with no positive events were required for the WT control.

### 2.6. PDX Generation and Therapy Testing

The UA was mechanically disaggregated with the VWR Pellet Mixer (VWR International, Radnor, PA, USA) and through a 30 G needle. Fetal bovine serum (Invitrogen, Carlsbad, CA, USA) was added to facilitate disaggregation. A Matrigel matrix (BD Biosciences, Franklin Lakes, NJ, USA) was added in a 1:2 ratio and kept on ice until use. Then, 50 µL of this mix was injected into the uteri of 8-week-old female SCID-beige mice (Janvier Labs; Le Genest Saint-Isle, France).

For implantation of the processed UA, mice were housed and maintained under specific-pathogen-free conditions, and procedures were performed in accordance with institutional guidelines and approved by the Use Committee for Animal Care from the Universidad de Santiago de Compostela. Aseptic procedures were followed for all surgeries. Each 8-week-old female SCID-beige mouse (Janvier Labs, Le Genest Saint-Isle, France) was anesthetized with 2% isoflurane/air (Isoflo, Esteve Farma, Carnaxide, Portugal) and kept under anesthesia for the entire procedure. The area was prepared for sterile surgery by shaving off the fur and scrubbing with a betadine solution and sterile 4×4 gauze. A midline ventral incision was made in the skin and abdominal wall. Once open, the uterus was located and 50 µL of cell suspension (UA cell suspension in the Matrigel matrix (BD Biosciences) in a 1:2 ratio) was injected with a 30 G syringe. After the Matrigel was allowed to solidify, the syringe was removed and the uterus was returned to the abdominal cavity. The wound was closed with Ethicon VICRIL (Johnson & Johnson International, Diegem, Belgium) sutures. Buprenodale (Dechra, Dales Pharmaceuticals, Keighley Road, Skipton, UK) was used for postoperative analgesia.

The orthotopic tumors that developed in the uteri were minced into small pieces and placed subcutaneously in the flanks of 8-week-old female SCID-beige mice (*n* = 11). For the preclinical studies, once the tumors reached a suitable size of 150–200 mm, they were randomly assigned to one of three groups and treated with placebo (methylcellulose 0.5%; Sigma; *n* = 4); CarboTaxol (carboplatin (50 mg/kg) or paclitaxel (20 mg/kg) combined therapy was administered intraperitoneally once every week for 4 weeks (*n* = 3); or BYL719, (50 mg/kg; Achemblock, Burlingame, CA; on the basis of the molecular alterations found in the UAs and a molecular tumor board consensus, as described in Section 2.4) previously dissolved in dimethylsulfoxide, was administered through oral gavage in methylcellulose 0.5% (Sigma) for 5 days on/2 days off until sacrifice (*n* = 4). All mice were sacrificed after the tumor diameter reached 15 mm, the tumor volume exceeded 1200 mm^3^, or the human endpoint criteria were met, according to the guidelines of Directive 2010/63/EU for the protection of animals used for scientific purposes, including assessment of appearance, body function, environment, and behavior. Tumor size was measured with calipers (Rohs) twice per week. Tumor volume was calculated according to the following formula: V = (W^2^ × L)/2 where V is the tumor volume, W is the tumor width, and L is the tumor length.

### 2.7. Statistical Analysis

Statistical analyses were performed in IBM SPSS Statistics 20, and graphs were generated in GraphPad Prism 5.0 (GraphPad Software, Inc., San Diego, CA, USA). Two-tailed Mann–Whitney U test or Kruskal–Wallis test was used to evaluate the differential cfDNA, ctDNA, and CTC levels among clinical groups. The Pearson correlation test was performed to determine the relationship between quantitative experimental and clinical variables. Associations between clinicopathologic features and the presence of ctDNA or CTCs were examined with the chi-square test (Fisher’s exact test). A *p*-value < 0.05 was set as the level of statistical significance.

## 3. Results

### 3.1. Clinicopathologic Characteristics of the EC cohort

Sixty patients who were diagnosed with EC and had to undergo surgery were included in the study. The clinicopathologic information (Table 1) indicated that 71.7% were endometrioid carcinomas (EEC) and 28.3% were non-endometrioid carcinomas (NEEC), with serous histology being the most frequent NEEC subtype. Most patients included in the study were diagnosed with EC as a primary lesion, whereas seven patients showed recurrent disease at the time of sample collection. In addition, 40% of tumors were of grade 3 (11 EEC and 13 NEEC), and 60% of them were classified as high-risk tumors after surgery (19 EEC and 17 NEEC) according to histology, tumor grade, and the extent of myometrial infiltration [12].

### 3.2. UA Sequencing to Characterize EC

**Figure 2:**
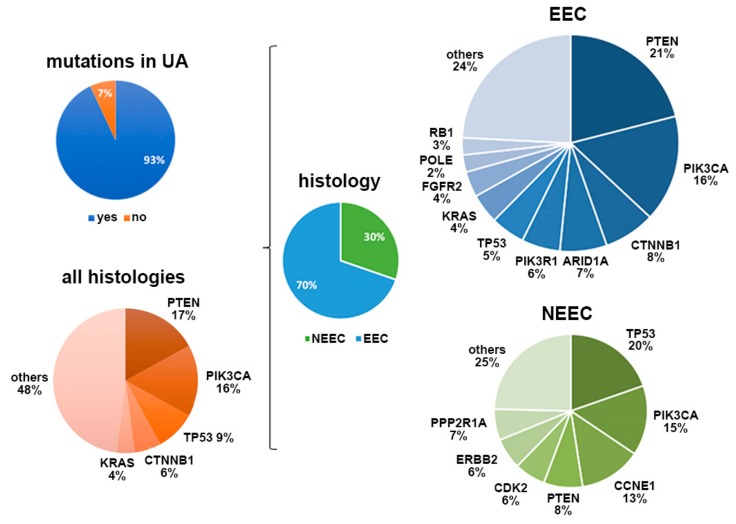
Summary of the most prevalent altered genes in UAs and their distribution according to tumor histology. UA, uterine aspirate; EEC, endometrioid carcinomas; NEEC, non-endometrioid carcinomas.

Targeted sequencing of the UAs from each of the 60 recruited patients was performed with the Oncomine Comprehensive Panel v3, as indicated in the Materials and Methods section. This panel included 87 hotspot regions, 48 full-length genes, 43 copy number regions in specific genes, and 188 gene fusions for 51 genes, all of which are frequently mutated in cancer. Indeed, most of these genetic and genomic regions have been observed to be altered in EC [13]. A sequence analysis revealed the presence of at least one genetic aberration in 56 of the 60 samples (93%), point mutations were identified in 52 patients (86.6%), and copy number variations (CNVs) were identified in 12 patients (23.2%) (Figure 2, Table S1). As expected, most of the EEC cases (38 of 43 cases) showed point mutations, except for one case in which a CNV was also found. In contrast, in NEEC cases (n = 17), point mutations and CNVs were detected in 14 and 11 cases, respectively, and eight NEEC cases showed both molecular alterations. Indeed, most of the identified genetic alterations were in *PTEN* (17.4%), *PI3KCA* (15.6%), *TP53* (9.2%), *CTNNB1* (5.5%), and *KRAS* (4.12%) genes, in line with the EC genomic landscape described in other studies [14] (Figure 2). In addition, as expected, *TP53* mutations were strongly associated with non-endometrioid histology (*p* < 0.001) and with high-grade carcinomas (*p* < 0.001), whereas *PI3KCA*, *CTNNB1*, *PTEN*, and *KRAS* were mainly found altered in endometrioid carcinomas. Likewise, *POLE*-positive cases were detected in only endometrioid tumors as *ARID1* mutations, which mainly appeared in endometrioid histology. Furthermore, 14 of the 60 (23.3%) UAs showed more than one point mutation in the same genes, mostly in *PTEN* and *PIK3CA*, thus supporting the presence of intratumor genetic heterogeneity (ITH) in these tumors (Table S1). Most of these tumors had endometrioid histology (*n* = 12) rather than non-endometrioid histology (*n* = 2).

### 3.3. cfDNA and ctDNA are Associated with Risk Factors in EC

**Figure 3:**
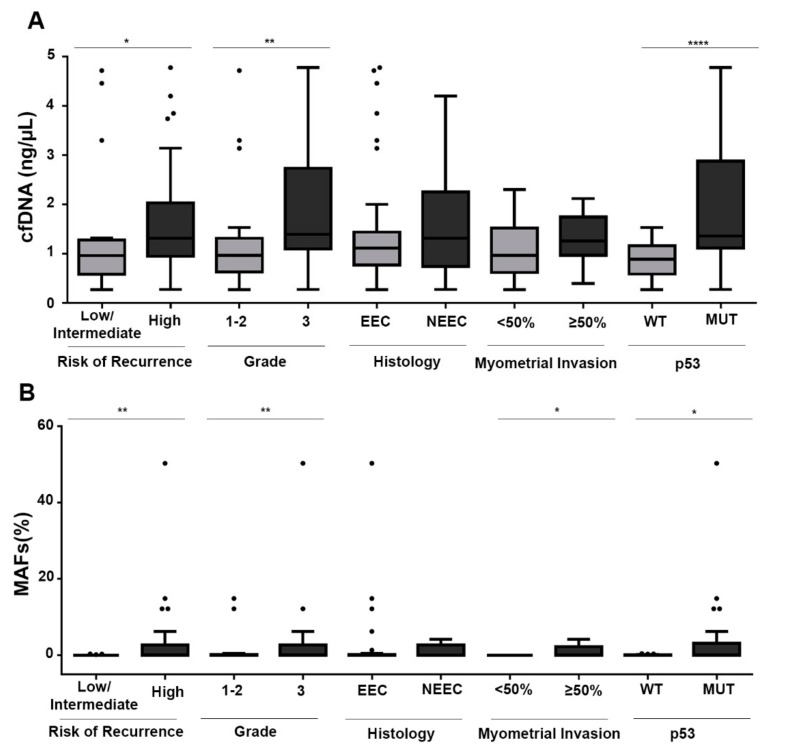
(**A**) Cell-free DNA (cfDNA) levels at surgery, grouped according to tumor grade, myometrial infiltration, risk of recurrence, and p53 status. (**B**) MAF (mutated allelic frequency) levels of the patient-specific point mutations, analyzed by ddPCR and grouped according to tumor grade, myometrial infiltration, risk of recurrence, and p53 status (n = 51). p53 was considered either mutant or wild type on the basis of immunohistochemistry analysis of primary tumors and UA sequencing. Mann–Whitney U tests were used to calculate the *p*-values. * *p* < 0.05, ** *p* < 0.01, **** *p* > 0.0001.

After the tumor mutational landscape was analyzed in UAs, we assessed the cfDNA cargo and patient-specific mutations in the ctDNA of each patient. The cfDNA concentration and quality were evaluated with Qubit fluorometry and TapeStation technology (Figure S2A). The cfDNA concentration at surgery ranged between 0.26 and 4.8 ng/µL and had a mean value of 1.5 ng/µL. Notably, cfDNA levels were significantly higher in patients with grade 3 tumors and those with a high risk of recurrence (Figure 3). Likewise, non-endometrioid and recurrent tumors displayed higher cfDNA levels, although the results did not reach statistical significance (Figure 3A, Table 2). Moreover, when the molecular profiles of tumors were analyzed together with the cfDNA levels, the presence of p53 mutations (determined in UAs through next-generation sequencing (NGS) or in primary tumors through immunohistochemistry) were clearly associated with higher levels of cfDNA (Figure 3A). Together, these results indicated increased DNA release into the circulation in patients with aggressive high-risk endometrial tumors.

We next used ddPCR analysis of the isolated cfDNA to explore the presence of the patient-specific mutations previously identified in the UAs. Briefly, for each patient, we selected the mutations found at higher frequency on the basis of the NGS study of UAs, and we validated the corresponding ddPCR assays for the detection of low mutant allele frequency (MAF). The LOD for point mutations ranged from 0.1% to 0.03%, whereas for CNVs, the general LOD varied from 8% to 20% (depending on the copy number identified in the UAs) (Figure S2B–D). An efficient ddPCR assay was successfully developed for 51 patients. In the remaining patients, ctDNA levels could not be assessed, owing to technical limitations in the design of the ddPCR assay or the absence of genetic alterations in UAs. Patients positive for ctDNA in the ddPCR were defined as those showing at least one point mutation or gene amplification in their cfDNA. Overall, ctDNA positivity was observed in 41.2% (21 of 51) of cases in which cfDNA could be assessed by ddPCR. Notably, 56.3% (18 of 32) of the high-risk tumors analyzed were positive for ctDNA at the time of surgery, whereas only 15.8% (3 of 19) of the low/intermediate-risk tumors were positive for ctDNA at surgery. Regarding clinical variables, patients with high myometrial infiltration had the highest rate of positive cases (58.6% positive, *p* = 0.008 Fisher’s test) (Table 2).

When disaggregated according to risk of recurrence and type I and type II EC, mainly high-risk EECs (68.7%; 11 of 16) were found to be positive for ctDNA, whereas NEECs (43.75%; 7 of 16) and low/intermediate-risk endometrioid carcinomas (15.8%; 3 of 19) were found to be positive for ctDNA. More interestingly, significantly higher positivity rates for ctDNA were associated with higher levels of cfDNA, suggesting a higher sensitivity of the ddPCR-based assay in this group of patients (*p* = 0.04, according to Mann–Whitney U tests; Figure S2E). In addition, the levels of ctDNA (mutant allelic frequency, MAFs) were higher in tumors with a higher grade or myometrial infiltration (Figure 3B). Among EECs, MAF levels were significantly higher in patients at high risk of recurrence (Figure S3A). Additionally, a significant correlation with the myometrial infiltration, grade, size, and lymph node affectation was also observed when total ctDNA was analyzed (product of cfDNA concentration and MAF ratio) (Figure S3B). Thus, these results indicated that ctDNA presence is clearly linked to a more aggressive disease and may provide a valuable tool for the detection of patients at risk of recurrence. In fact, preliminary results during the post-surgery follow-up by ctDNA analysis enabled us to detect three cases of recurrence after 12 months (Patient #24, serous carcinoma), 7 months (Patient #41, endometrioid carcinoma), and 18 months (Patient #971, endometrioid carcinoma). Patient #24 showed ctDNA at baseline and progression, whereas the other two patients showed positive ctDNA only at progression (Figure S4).

### 3.4. Additional Value of CTC Enumeration in EC

**Figure 4:**
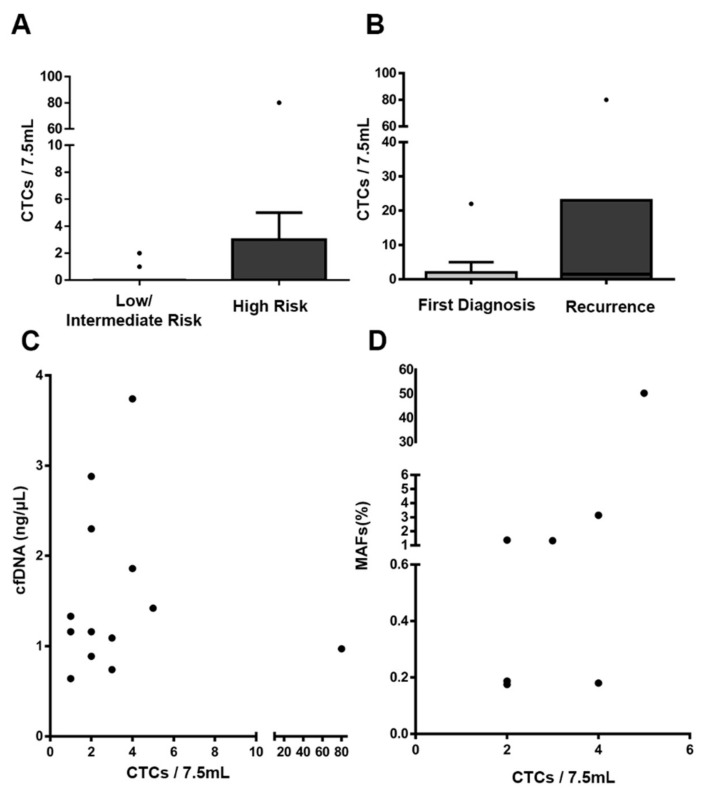
CTC enumeration with the CellSearch system. (**A**–**B**) CTC levels according to the risk of recurrence and the disease status at sample collection (first diagnosis versus recurrent disease). (**C**) Correlation between cfDNA concentration and CTC count. (**D**) Correlation between ctDNA levels (MAFs) and CTC count; n = 33 patients for ctDNA and CTC comparisons.

We then analyzed the CTC population in a subset of patients by using the reference technology for CTC enumeration (CellSearch technology). Thirty-six patients (25 high-risk and 11 low/intermediate-risk patients) were analyzed, including cases of both EEC (n = 23) and NEEC (n = 13), as detailed in Table 2. Overall, 38.9% (14 of 36) of patients were positive for CTC detection (1–80 CTCs/7.5mL) and 73% showed low CTC levels (fewer than five CTCs). The percentage of positivity was slightly lower in EEC (34.8%; 8 of 23) than NEEC (46.1%; 6 of 13) (Table 2). Higher rates of positivity were found on high-risk and recurrent disease, although they did not reach significance (Table 2, Figure 4A,B). In contrast, when CTCs and ctDNA results were considered together (n = 33), we found that only seven (21%) patients were positive for both circulating markers, thus suggesting that both strategies can provide complementary information. In fact, 80% of the high-risk tumors were positive for CTCs and/or ctDNA. Interestingly, we also observed a correlation between CTC counts and MAF levels in positive cases for both analyses and a trend toward higher levels of CTCs in patients with higher levels of cfDNA, although the results did not reach statistical significance (Figure 4C,D).

These results confirmed the additional value of CTC detection for risk evaluation, and the complementary ability of ctDNA and CTC analyses to provide a global overview of all risk factors influencing patients’ disease evolution after surgery.

### 3.5. UAs for the Selection of Personalized Therapies and as a Feasible Alternative to Generate PDX Models

**Figure 5:**
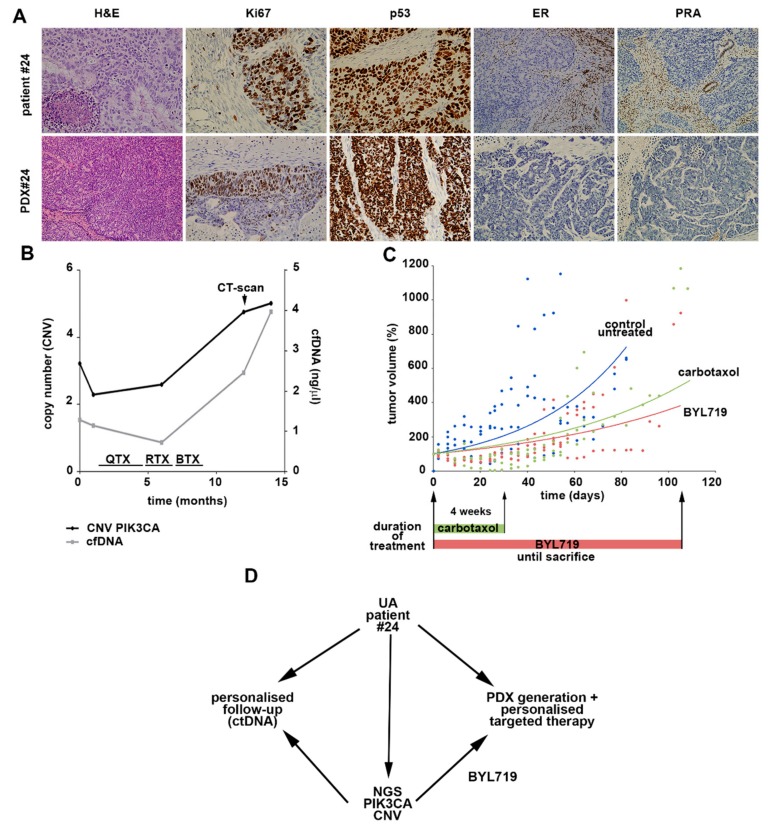
Patient-derived xenograft (PDX) generation from uterine aspirate (UA) of Patient #24 as a preclinical model to test targeted therapies. (**A**) Comparison of histology and immunohistochemistry between the patient primary tumor and the PDX generated from the UA collected at surgery. (**B**) cfDNA and ctDNA level dynamics during disease evolution. QTX: chemotherapy; RTX: radiotherapy; BTX: brachytherapy. (**C**) PDX tumor growth evolution in response to carboplatin/paclitaxel (weekly intraperitoneal injection for 4 weeks, n = 3), BYL719 (daily oral gavage; n = 4), or control (methyl cellulose daily oral gavage; n = 4) treatment. (**D**) Summary of the combined liquid biopsy strategy to achieve personalized treatment for the patients with EC included in our study.

We completed a personalized pipeline to improve EC clinical management by exploring the clinical utility of UAs for the selection and validation of targeted therapies in EC. For this purpose, we exploited the value of the UA as a liquid biopsy to characterize the overall mutational landscape of EC together with its potential as a tool to develop PDX models, which recapitulate the heterogeneity of primary tumors. Through this procedure, we selected Patient #24, diagnosed with serous NEEC and presenting with ctDNA at the time of surgery and recurrent disease 12 months after debulking surgery, with elevated levels of ctDNA (Figure 5B). An orthotopic model was generated by orthotopic injection of the UA into the uteri of immunodeficient mice. Histology and immunohistochemistry analyses demonstrated that the UA PDX model reliably reproduced the histologic and molecular characteristics of the original patient carcinoma (Figure 5A). After generation of the orthotopic tumor, subcutaneous passage was conducted in a series of mice (n = 11) for amplification and evaluation of therapy efficacy. The mutational analysis of the UA was used to screen for specific therapies with bioinformatic tools, thus resulting in the selection of the orally bioavailable BYL719 (Alpelisib) inhibitor as a therapy for targeting the altered PIK3CA (phosphatidylinositol-4,5-bisphosphate 3-kinase catalytic subunit alpha) pathway in Patient #24. We then designed a preclinical study to evaluate the efficacy of this PI3KCA inhibitor compared with standard therapy based on carboplatin–paclitaxel. Mice were equally distributed in three groups: a control untreated group, a control group treated with the standard carboplatin/paclitaxel therapy (weekly intraperitoneal injection for 4 weeks), and a group treated with daily oral administration of BYL719 (scheme in Figure 5C).

As shown in Figure 5C, the PDX model originating from the UA of serous carcinoma showed similar responses to both BYL719 and the standard therapy compared to the untreated group, although tumor growth was observed after termination of the four cycles of the standard therapy, whereas re-growth was found in the BYL719 group. Similar results were observed with a high-risk endometrioid PDX model that was generated from Patient #7 and was also characterized as having a *PIK3CA* mutation from UA sequencing and an additional mutation in *KRAS*. BYL719 treatment was compared with untreated controls and standard therapy (Figure S5). These results reinforced the clinical value of UAs in (i) characterizing the molecular landscape of endometrial carcinomas, (ii) identifying therapies targeting specific molecular pathways in specific patients with EC, and (iii) generating suitable preclinical models to validate the identified personalized therapies (Figure 5D).

## 4. Discussion

Patients normally have good prognosis if diagnosis occurs in early stages of EC. However, some patients experience recurrence after surgery, and this recurrence is not predictable with the current risk classification systems. Clinical management of the risk of recurrence remains an unsolved issue that is probably associated with tumor heterogeneity and early tumor dissemination [15]. Therefore, new strategies must be developed to improve risk stratification, therapy selection, and monitoring of this disease. In this sense, studies based on liquid biopsy may be essential to achieve more precise clinical management in EC. The advantages of using liquid biopsies rather than tissue samples are clear: the samples are easy to obtain, provide information in real time, and improve the understanding of tumor heterogeneity [16]. Nevertheless, despite the many technical improvements in liquid biopsies in other tumors during the past two decades, information regarding the clinical benefit of using these alternative biopsies to manage patients with EC is quite limited [6].

Our study performed the first combined characterization of UAs, ctDNA, and CTCs to comprehensively explore the value of liquid biopsies for personalizing EC treatment. To do so, we determined the genomic landscape of UA samples obtained at surgery in a relevant cohort of patients. The samples recapitulated the mutational patterns found in tissue samples from ECs and had the additional advantages of being minimally invasive and representative of the molecular heterogeneity of primary carcinomas. We were able to detect genetic alterations in 93% of the UA samples analyzed with targeted sequencing. *PI3KCA*, *PTEN*, *TP53*, *CTNB1*, and *KRAS* were the genes most frequently mutated in our cohort, in accordance with the genomic pattern previously described in primary carcinomas [10,14,17,18]. Moreover, the genomic signatures reliably reproduced the molecular classification of the histology subtypes: serous UA was mainly characterized by *TP53* point mutations and CNVs, whereas endometrioid UA showed a broader spectrum of mutations, primarily *PI3KCA* and *PTEN* alterations [17]. Importantly, 23.3% of UAs showed intratumor genetic heterogeneity (ITH), considered as the presence of more than one point mutation in the same genes, mostly in *PTEN* and *PIK3CA*. These findings reinforced our previous observations [10] regarding the use of sequencing analysis of UAs to capture ITH as an alternative diagnostic biopsy to aid in the selection of more specific treatments.

In addition to providing a valuable tool to better understand the biology of each endometrial tumor, the characterization of UAs also facilitated the translation of a personalized approach to relevant clinical blood samples through the analysis of patient-specific mutations with cfDNA. The levels of cfDNA in EC have been studied [19,20]; higher cfDNA levels have been reported in serum samples from patients with EC than in healthy control individuals and patients with benign gynecologic disorders, although the cfDNA levels are independent of tumor stage or grade [19]. In our study, including both endometrioid and non-endometrioid tumors, cfDNA levels at surgery were significantly higher in high-risk tumors. Therefore, cfDNA levels have potential as a prognostic factor, although long-term follow-up of patients will be required to definitively demonstrate the clinical value.

Importantly, the rate of positive cases for ctDNA at surgery was 41.2%, and this rate was higher in high-risk tumors. Even though these tumors were not characterized for their DNA release and not all mutations found in UA sequencing were followed by ddPCR, the detection rate presented in this article with our targeted approach was higher than that previously described with other technologies, such as NGS [9]. Moreover, the rate of ctDNA-positive cases found correlated with myometrial and lymphovascular infiltration and with histology grade, in line with recent data [9]. In addition, our results also demonstrated the feasibility of ctDNA monitoring in patients with EC to identify the presence of a more aggressive disease, as well as the utility of assessment of ctDNA in combination with cfDNA as potential risk factors for identifying patients with higher risk of recurrence. In fact, the levels of ctDNA at surgery and during follow-up were associated with the recurrence of the disease in three of the patients included in the study. As mentioned above, long-term follow-up of the patients included in the study will be necessary to confirm the clinical value of ctDNA determination in improving the risk classification of patients with EC. In this regard, Pereira et al., by analyzing a retrospective cohort of gynecologic tumors (ovary and endometrial tumors), have found lower survival rates in patients with detectable ctDNA levels at surgery [21].

CTC enumeration showed similar, but slightly higher, rates of positivity than that reported in previous studies in EC analyzed with the CellSearch system (probably associated with the specific characteristics of the different cohorts) [7,22]. Notably, we found that in patients with detectable ctDNA and CTCs, the levels of both markers were correlated, and by considering the presence of CTCs and/or ctDNA, most high-risk tumors (80%) could be identified. In line with our results, previous studies have also indicated discrepancies between CTC presence and the release of ctDNA into the bloodstream, owing to the different natures of both circulating tumor markers, thus suggesting that this method provides complementary, clinically valuable information in EC [23,24].

Finally, our study also pioneered the use of UAs in selecting targeted therapies based on the patient-specific genomic landscape, and generating PDX models as an individualized approach to evaluate their efficacy. We were able to generate PDX models closely resembling the patient tumor characteristics and reproducing the molecular and histologic features. The preclinical study targeting the identified PI3KCA-activating alteration confirmed the activity of BYL719 (a specific PI3KCA inhibitor) and demonstrated the clinical value of our personalized pipeline based on liquid biopsies to address therapeutic alternatives.

## 5. Conclusions

In conclusion, the global liquid biopsy strategy presented in this work, in which sequencing of UAs from individual patients with EC was complemented by cfDNA, ctDNA, and CTC analyses, confirmed the clinical utility of the management of patients with EC, including an accurate risk classification and the potential selection of more personalized therapies. This non-invasive approach clearly provides a valuable tool for precision medicine in EC and can probably be extended to other gynecologic tumors.

## Figures and Tables

**Table 1:** Clinicopathologic characteristics of the cohort of endometrial cancer (EC).

Feature	Low/Intermediate Risk n = 24	High Risk *n* = 36	Total *n* = 60
AGE (38–88 y/o*)			
<66 y/o	10 (41.67%)	14 (38.89%)	24 (40.00%)
≥66 y/o	14 (58.33%)	22 (61.11%)	36 (60.00%)
**Time of diagnosis**			
Recently diagnosed	22 (91.67%)	31 (86.11%)	53 (88.33%)
Recurrence	2 (8.33%)	5 (13.88%)	7 (11.66%)
**Histology**			
Endometrioid	24 (100.00%)	19 (52.78%)	43 (71.67%)
Non-endometrioid	0 (0.00%)	17 (47.22%)	17 (28.33%)
Histologic grade			
Grade 1	15 (62.50%)	5 (13.89%)	20 (33.33%)
Grade 2	8 (33.33%)	7 (19.44%)	15 (41.66%)
Grade 3	1 (4.17%)	24 (66.66%)	25 (69.44%)
**Figo stage**			
I	23 (95.83%	13 (36.11%)	36 (60.00%)
II	1 (4.17%)	9 (25.00%)	10 (16.67%)
III	0 (0.00%)	11 (30.56%)	11 (18.33%)
IV	0 (0.00%)	3 (8.33%)	3 (5.00%)
**Myometrial invasion**			
<50%	15 (62.50%)	10 (27.78%)	25 (41.67%)
≥50%	9 (37.50%)	25 (69.44%)	34 (56.67%)
Unknown	0 (0.00%)	1 (2.78%)	1 (1.67%)
LVSI**			
No	13 (54.17%)	18 (50.00%)	31 (51.67%)
Yes	3 (12.50%)	11 (30.56%)	14 (23.33%)
Unknown	8 (33.33%)	7 (19.44%)	15 (25.00%)

* y/o: years old; ** LVSI: lympho-vascular space involvement.

**Table 2:** Levels of cfDNA, circulating tumor DNA (ctDNA), and circulating tumor cells (CTCs) according to the clinicopathologic features of patients with EC.

Feature	cfDNA Mean ± SEM** (ng/µL)	*p*	ctDNA-Positive Patients	*p*	CTCs/7.5 mL-Positive Patients	*p*
**Histology**						
Endometrioid	1.44 ± 0.17		14/35 (40.00%)		8/23 (34.78%)	
Non-endometrioid	1.65 ± 0.28	0.28	7/16 (43.75%)	1.0	6/13 (46.15%)	0.72
**Histologic grade**						
Grade 1/2	1.17 ± 0.15		8/27 (29.63%)		5/27 (18.52%)	
Grade 3	2.02 ± 0.26	**0.003**	13/24 (54.17%)	**0.049**	9/19 (65.89%)	0.18
**Figo stage**						
I/II	1.42 ± 0.17		12/35 (34.29%)		6/22 (27.27%)	
III/IV	1.79 ± 0.38	0.38	7/12 (58.33%)	0.18	5/9 (55.55%)	0.21
**Myometrial invasion**						
<50%	1.30 ± 0.21		4/21 (19.05%)		4/12 (33.33%)	
≥50%	1.67 ± 0.21	0.08	17/29 (58.62%)	**0.008**	10/23 (43.48%)	0.72
**LVSI**						
No	1.38 ± 0.18		10/27 (37.04%)		5/15 (33.33%)	
Yes	2.15 ± 0.41	0.07	7/13 (53.85%)	0.49	5/10 (50.00%)	0.44
**Risk of recurrence**						
Low/intermediate	1.24 ± 0.23		3/19 (15.79%)		2/11 (18.18%)	
High	1.65 ± 0.19	**0.017**	18/32 (56.25%)	**0.007**	12/25 (48.00%)	0.14
**Time of diagnosis**						
Recently diagnosed	1.50 ± 0.16		17/45 (37.78%)		10/30 (33.33%)	
Recurrence	1.44 ± 0.23	0.45	4/6 (66.67%)	0.21	4/6 (66.67%)	0.11

* LVSI: Lympho-vascular space involvement; ** SEM: standard error of the mean. Bold numbers represent statistically significant differences between experimental groups.

## Supplementary Materials

The following are available online at https://www.mdpi.com/2077-0383/9/2/585/s1, Figure S1. Representative examples of CTCs found in patients with EC (*n* = 36) using the CellSearch technology. Round-oval, DAPI+, CD45−, and CK+ cells were considered CTCs. Figure S2. (A) Fragment distribution of the cfDNA extracted from the plasma of patients with EC analyzed with TapeStationHS1000 (Agilent). (B) Representative example of the correlation between the expected and obtained ratios in a LOD ddPCR assay to detect a point mutation in PIK3CA (R88Q), LOD = 0.03%. (C) Correlation between the expected number of mutated copies and that obtained after ddPCR to detect a point mutation in PIK3CA (R88Q). (D) Representative example of the LOD to detect CNV of the PIK3CA gene, LOD = 8.00%. Figure S3. (A) ctDNA levels (MAFs) detected in EEC and grouped according to tumor grade, myometrial infiltration, risk of recurrence, and p53 status (*n* = 34). (B) ctDNA levels represented as ng/μL in EC and grouped according to tumor grade, myometrial infiltration, risk of recurrence, and p53 status (*n* = 51). Figure S4. cfDNA and ctDNA levels during disease evolution in two patients with EC that progressed after surgery. (A) Patient #41 (EEC) progressed 7 months after surgery. A point mutation in CTNNB1 (p.T41A) was found in the UA and then monitored at surgery and progression (PD) with ddPCR. (B) Patient #971 (EEC) progressed 18 months after surgery. A point mutation in BRAF (p. N581S) was found in the UA and then monitored at surgery and progression (PD) with ddPCR. RTX: radiotherapy; BTX: brachytherapy. Figure S5. PDX generation from UA as a preclinical model to test targeted therapies in Patient #7. (A) Hematoxylin and eosin staining showing the same type of histology in the primary tumor and the PDX. (B) PDX tumor growth evolution in response to the treatments. Twelve mice were distributed in three groups: a control untreated group, a control group with standard therapy of carboplatin/paclitaxel (weekly intraperitoneal injection for 4 weeks), and a group with BYL719 orally administered daily for 2 weeks, according to the literature. In the PDX #24 model, continuous administration of BYL719 was maintained until sacrifice because of the promising results with the BYL719 treatment (Figure 5). Table S1. List of tumor-specific mutations identified by NGS in the UAs obtained from patients with EC at surgery.
