# Supplementary material for: Genomic Profiling of Uterine Aspirates and cfDNA as an Integrative Liquid Biopsy Strategy in Endometrial Cancer
Source: J Clin Med. 2020 Feb 21;9(2):585. doi: 10.3390/jcm9020585 (PMC7073542; doi:10.3390/jcm9020585)
Supplement: Supplementary file 1 [file jcm-09-00585-s001.pdf]

Table S1. List of tumor-specific mutations identified by NGS in the UAs obtained from patients with EC at surgery.

GENE	ID	NUCLEOTIDE	PROTEIN	GAIN/LOSS	FREQUENCY (MAFs)
CTNNB1	**EndoBL005**	c.110C>T	p.Ser37Phe	Gain	**25.35**
PIK3CA		c.1258T>C	p.Cys420Arg	Gain	**22.57**
FGFR2		c.755C>G	p.Ser252Trp	Gain	**20.42**
PIK3CA	**EndoBL007**	c.263G>A	p.Arg88Gln	Gain	**43.91**
KRAS		c.35G>A	p.Gly12Asp	Gain	**19.87**
PIK3CA	**EndoBL009**	c.113G>A	p.Arg38His	Gain	**9.26**
PIK3CA		c.3074C>G	p.Thr1025Ser	Gain	**19.12**
KRAS		c.35G>A	p.Gly12Asp	Gain	**21.21**
TP53	**EndoBL010**	c.613T>G	p.Y205D	Loss	**19.66**
PIK3CA		c.323G>A	p.Arg108His		**53.23**
KRAS	**EndoBL013**	c.175G>A	p.Ala59Thr	Gain	**25.2**
MYC	**EndoBL015**	amplification		Gain	**5.41**
CCNE1		amplification		Gain	**16.21**
TP53		c.524G>A	p.R175H	Loss	**10**
KRAS		amplification		Gain	**8.56**
CTNNB1	**EndoBL019**	c.98C>T	p.Ser33Phe	Gain	**48.72**
AKT1		c.49G>A	p.Glu17Lys	Gain	**55.81**
PPP2R1A	**EndoBL022**	c.770G>T	p.W257L	Gain	**45.37**
TP53		c.838A>G	p.R280G	conflicting interpretations of pathogenicity	**56.97**

PIK3CA	**EndoBL024**	copy number mean (8.66)		Gain	
KRAS	**EndoBL026**	c.437C>T	p.Ala146Val	Gain	**26.15**
PIK3CA	**EndoBL028**	c.1633G>A	p.Glu545Lys	Gain	**30.05**
PTEN		c.389G>C	p.Arg130Pro	Loss	**44.91**
CTNNB1	**EndoBL029**	c.110C>T	p.Ser37Phe	Gain	**28.9**
PIK3CA		c.3127A>G	p.Met1043Val	Gain	**30.47**
ESR1		c.1610A>C	p.Tyr537Ser	Gain	**33.03**
PTEN		c.511C>T	p.Gln171Ter	Loss	**27.29**
FGFR2		c.755C>G	p.Ser252Trp	Gain	**31.97**
POLE	**EndoBL031**	c.857C>G	p.Pro286Arg	Loss	**14**
ARID1A		c.5299G>T	p.Glu1767Ter	Loss	**36.46**
PIK3CA		c.333G>T	p.Lys111Asn	Gain	**25**
PIK3CA		c.1357G>A	p.Glu453Lys	Gain	**41.3**
PIK3CA		c.3141T>G	p.His1047Gln	Gain	**38.13**
ATM		c.748C>T	p.Arg250Ter	Loss	**38.94**
RB1		c.7348C>T	p.Arg2450Ter	Loss	**36.51**
MLH1	**EndoBL034**	c.2035G>T	p.Glu679Ter	Loss	**16.78**
CTNNB1		c.94G>A	p.Asp32Asn	Gain	**6.26**
PIK3CA		c.263G>A	p.Arg88Gln	Gain	**15.96**
PIK3R1		c.1519G>T	p.Glu507Ter	Loss	**16.15**
PTEN		c.373A>G	p.Lys125Glu	Loss	**15.41**
PTEN		c.462C>A	p.Phe154Leu	Loss	**16.51**
ATM		c.742C>T	p.Arg248Ter	Loss	**15.02**
POLE		c.857C>G	p.Pro286Arg	Loss	**16.8**
RB1		c.709G>T	p.Glu237Ter	Loss	**13.78**
SLX4		c.4523C>T	p.Ser1508Leu	Unknown	**14.98**

CTNNB1	**EndoBL_MDA_1**	c.98C>G	p.Ser33Cys	Gain	**34.48**
PIK3CA		c.1634A>C	p.Glu545Ala	Gain	**25**
PTEN	**EndoMDA044**	c.881_882delGT	p.Ser294fs	Loss	**40.66**
KRAS		c.34G>A	p.Gly12Ser	Gain	**39.4**
NRAS	**Endo VH02**	c.182A>G	p.Gln61Arg	Gain	**12.61**
TP53		c.610G>T	p.Glu204Ter	Loss	**28.47**
PIK3CA	**Endo VH14**	c.3129G>T	p.Met1043Ile	Gain	**37.96**
RICTOR		copy number mean (5.78)		Gain	**5%:5.06. 95%:6.6**
TP53		c.722C>G	p.Ser241Cys	Loss	**74.55**
PPP2R1A		c.767C>A	p.Ser256Tyr	Gain	**29.34**
PTEN	**Endo VH30**	c.362C>A	p.Ala121Glu	Loss	**40.55**
PTEN		c.638delC	p.Pro213fs	Loss	**43.05**
RB1		c.1001G>T	p.Arg334Ile	Unknown	**12.06**
TP53		c.838A>G	p.Arg280Gly	Loss	**14.09**
CCNE1		copy number mean (19.69)		Gain	**5%:16.82. 95%:23.04**
PTEN	**EndoVH33**	c.389G>C	p.Arg130Pro	Loss	**20.4**
ARID1A	**Endo VH35**	c.5965C>T	p.Arg1989Ter	Loss	**43.57**
CTNNB1		c.110C>T	p.Ser37Phe	Gain	**39.61**
PIK3CA		c.3129G>T	p.Met1043Ile	Gain	**40.1**
FBXW7		c.1972C>T	p.Arg658Ter	Loss	**42.61**
PIK3R1		c.1042C>T	p.Arg348Ter	Loss	**87.16**
PTEN		c.19G>T	p.Glu7Ter	Loss	**43.37**
CREBBP		c.3955C>T	p.Arg1319Ter	Loss	**34.18**
FGFR3	**964**	copy number mean (5.77)		Gain	**5%:5.19, 95%:6.42**

ARID1A	**970**	c.5164C>T	p.Arg1722Ter	Loss	**4.35**
CTNNB1		c.110C>T	p.Ser37Phe	Gain	**41.12**
PIK3CA		c.331A>G	p.Lys111Glu	Loss	**37.92**
FBXW7		c.1393C>T	p.Arg465Cys	Gain	**3.7**
BRAF	**971**	c.1742A>G	p.Asn581Ser	Gain	**45.6**
WHSC1L1(1) - FGFR1(2)		Gene fusion		Gain	**2927 counts**
PTEN		c.916delA	p.Ile306Ter	Loss	**50.17**
RB1		c.2087_2090delGA GA	p.Arg696fs	Loss	**13.15**
POLE	**1002**				
PIK3CA	**1009**	c.317G>T	p.Gly106Val	Gain	**34.8**
CDK2		copy number mean (5.64)		Gain	**5%:5.02, 95%:6.34**
NOTCH3		c.2538_2539insT	p.Asp847Ter	Loss	**3.5**
MTOR	**EndoBL053**	c.6644C>T	p.Ser2215Phe	Gain	**36.29**
PIK3CA		c.3129G>T	p.Met1043Ile	Gain	**38.27**
PTEN		c.144C>A	p.Asn48Lys	Loss	**39.97**
PTEN		c.389delG	p.Arg130fs	Loss	**40.61**
KRAS		c.38G>A	p.Gly13Asp	Gain	**39.29**
ARID1A	**EndoBL056**	c.4003C>T	p.Arg1335Ter	Loss	**22.31**
PIK3CA		c.1636C>A	p.Gln546Lys	Gain	**26.13**
PIK3R1		c.1669C>T	p.Arg557Ter	Loss	**37.66**
PTEN		c.367C>G	p.His123Asp	Loss	**35.91**
PTEN		c.388C>G	p.Arg130Gly	Loss	**34.72**
TP53		c.817C>T	p.Arg273Cys	Loss	**4.57**
TP53		c.583A>T	p.Ile195Phe	Loss	**4.11**
TP53		c.430C>T	p.Gln144Ter	Loss	**7.69**

ARID1A	**EndoBL057**	c.5890G>T	p.Glu1964Ter	Loss	**17.33**
CDK2		copy number mean (5.27)		Gain	**5%:4.22, 95%:6.58**
TP53		c.876delA	p.Glu294fs	Loss	**<5%**
ERBB2		copy number mean (5.43)		Gain	**5%:4.58. 95%:6.44**
PIK3CA	**EndoBL036**	c.263G>A	p.Arg88Gln	Gain	**24.06**
PIK3CA		c.353G>A	p.Gly118Asp	Gain	**19.53**
FGFR2		c.1647T>A	p.Asn549Lys	Unknown	**49.82**
ARID1A		c.482_495delTCGCCGCCGCCGCG	p.Val161fs	Unknown	**14.47**
PTEN	**EndoBL039**	c.81_82insT	p.Ile28fs	Loss	**37.04**
TP53		c.569C>T	p.Pro190Leu	Loss	**74.76**
PIK3CA	**EndoBL040**	c.1634A>G	p.Glu545Gly	Gain	**19.68**
PTEN		c.388C>G	p.Arg130Gly	Loss	**39.91**
FGFR1		c.495_497delTGA	p.Asp166del	Gain	**13.28**
PPP2R1A		c.547C>T	p.Arg183Trp	Gain	**42.38**
CTNNB1	**EndoBL041**	c.121A>G	p.Thr41Ala	Gain	**44.29**
RNF43		c.1585C>T	p.Arg529Trp	Unknown	**48.62**
PTEN		c.442G>A	p.Ala148Thr	Loss	**47.94**
NOTCH2	**EndoBL042**	c.5356C>T	p.Arg1786Ter	Loss	**25.83**
XPO1		c.1711G>A	p.Glu571Lys	Gain	**17.87**
PIK3CA		c.1030G>A	p.Val344Met	Gain	**35.36**
PIK3CA		c.3062A>G	p.Tyr1021Cys	Gain	**37.67**
FBXW7		c.979G>T	p.Glu327Ter	Loss	**36.67**
PIK3R1		c.1042C>T	p.Arg348Ter	Loss	**33.41**
PIK3R1		c.1156C>T	p.Arg386Ter	Loss	**33.87**
PTEN		c.101C>T	p.Ala34Val	Loss	**32.72**

PTEN		c.545T>G	p.Leu182Ter	Loss	**36.62**
PTEN		c.697C>T	p.Arg233Ter	Loss	**9.8**
ATM		c.9022C>T	p.Arg3008Cys	Loss	**32.12**
FANCI		c.1447G>T	p.Glu483Ter	Loss	**6.68**
TP53		c.1024C>T	p.Arg342Ter	Loss	**12.42**
NF1		c.532G>T	p.Glu178Ter	Loss	**34.37**
NF1		c.7348C>T	p.Arg2450Ter	Loss	**34.53**
MSH2		c.2272G>A	p.Asp758Asn	Unknown/potential pathogenic	**8.85**
POLE		c.4942G>A	p.Glu1648Lys	Loss	**34.15**
PTEN	**EndoBL049**	c.388C>T	p.Arg130Ter	Loss	**84.43**
ARID1A	**EndoMDA061**	c.1669C>T	p.Gln557Ter	Loss	**18.58**
PIK3CA		c.1633G>A	p.Glu545Lys	Gain	**23.27**
PTEN		c.203A>G	p.Tyr68Cys	Loss	**24.45**
PTEN		c.388C>G	p.Arg130Gly	Loss	**24.4**
FGFR2		c.755C>G	p.Ser252Trp	Gain	**21.6**
MAX		c.83A>G	p.His28Arg	Gain	**20.8**
MET		c.1038C>G	p.Phe346Leu	Unknown	**21.25**
TP53	**EndoVH046**	c.524G>A	p.Arg175His	Loss	**39.25**
CCNE1		copy number mean (7.92)		Gain	**5%:6.97, 95%:9.01**
PALB2	**EndoVH012**	c.656A>G	p.Asp219Gly	Unknown/conflicting interpretations of pathogenicity	**50.70**
ESR1(2) - CCDC170(8)	**EndoMDA062**	fussion	ESR1-CCDC170.E2C8	Gain	**186 copies**
PTEN	**EndoBL37**	c.389G>C	p.Arg130Pro	Loss	**69**
PIK3R1		c.978_982delGTCCT	p.Met326fs	Loss	**12.43**

TP53	**EndoBL63**	c.1024C>T	p.Arg342Ter	Loss	**68.88**
PPP2R1A		c.536C>G	p.Pro179Arg	Gain	**38.50**
PIK3CA		c.1049A>G	p.Asp350Gly	Gain	**43.47**
PIK3CA	**EndoBL65**	c.1357G>A	p.Glu453Lys	Gain	**51.48**
TP53		c.955A>T	p.Lys319Ter	Loss	**54.21**
TP53		c.518T>C	p.Val173Ala	Loss	**25.95**
ERBB2		copy number mean (4.89)		Gain	**5%:4.24, 95%:5.64**
PPP2R1A		c.536C>G	p.Pro179Arg	Gain	**38.97**
CTNNB1	**VH_1049**	c.110C>T	p.Ser37Phe	Gain	**16.80**
PTEN		c.469delG	p.Glu157fs	Loss	**24.13**
ARID1A	**VH_1056**	c.3283C>T	p.Gln1095Ter	Loss	**38.98**
PTEN		c.62delT	p.Phe21fs	Loss	**39.7**
PTEN		c.388C>T	p.Arg130Ter	Loss	**43.62**
FGFR2		c.1647T>A	p.Asn549Lys	Gain	**40.10**
ARID1A	**VH_605**	p.Gln505fs	c.1513_1514delCA	Loss	**26.28**
CTNNB1		p.Ser45del	c.133_135delTCT	Gain	**27.76**
PTEN		p.Glu73Ter	c.217G>T	Loss	**25.51**
CCND3	**VH_766**	copy number mean (5.28)		Gain	**5%:4.49, 95%:6.21**
MYC		copy number mean (29.63)		Gain	**5%:25.39, 95%:34.58**
CCNE1		copy number mean (4.79)		Gain	**5%:4.07, 95%:5.63**
AKT2		copy number mean (23.18)		Gain	**5%:19.86, 95%:27.05**
PPP2R1A		p.Pro179Arg	c.536C>G	Gain	**66.48**
PIK3R1	**VH_928**	p.Asp560His	c.1678G>C	Loss	**4.4**

TP53		p.Gly266Glu	c.797G>A	Loss	**67.33**
MSH2	**VH_959**	p.Arg680Ter	c.2038C>T	Loss	**49.27**
FBXW7		p.Arg224Ter	c.670C>T	Loss	**20.52**
PTEN		p.Gln245Ter	c.733C>T	Loss	**20.13**
CDKN1B		p.Pro137fs	c.410delC	Loss	**20.18**
MAP2K1		p.Pro124Ser	c.370C>T	Gain	**23.66**
ARID1A	**VH_1012**	p.Tyr930fs	c.2788delT	Loss	**36.08**
CTNNB1		p.Ser33Phe	c.98C>T	Gain	**5.7**
PIK3CA		p.Glu542Lys	c.1624G>A	Gain	**25.45**
FBXW7		p.Arg465Cys	c.1393C>T	Loss	**24.11**
PTEN		p.Glu43fs	c.128_129delAA	Loss	**32.06**
PTEN		p.Arg233Ter	c.697C>T	Loss	**25.4**
ERBB2		p.Gly776Val	c.2327G>T	Gain	**26.89**
PTEN	**VH_1077**	p.Arg130Gly	c.388C>G	Loss	**26.75**
RB1		p.Gln575Ter	c.1723C>T	Loss	**11.88**
TP53		p.Arg249Gly	c.745A>G	Loss	**13.22**
ARID1A	**VH_1087**	p.Arg693Ter	c.2077C>T	Loss	**20.37**
ARID1A		p.Ala2205fs	c.6611_6612insTCCTGGGCTTCCTAGAGGACAGCCTTGC	Loss	**8.18**
PIK3CA		p.Glu545Lys	c.1633G>A	Gain	**16.15**
PTEN		p.Asp92Asn	c.274G>A	Loss	**20.59**
KRAS		p.Gly12Asp	c.35G>A	Gain	**34.32**
CTNNB1	**VH_1093**	p.Asp32Val	c.95A>T	Gain	**23**
PIK3CA		p.Lys111Glu	c.331A>G	Gain	**37.25**
PIK3R1		p.Ile571fs	c.1710_1711insT	Loss	**43.31**

PIK3R1		p.Met582fs	c.1743delG	Loss	**43.37**
PTEN		p.Arg130Gln	c.389G>A	Loss	**32.75**
PTEN		p.Arg308fs	c.923delG	Loss	**57.59**
FBXW7	**VH_1099**	p.Arg465Cys	c.1393C>T	Loss	**29.85**
TP53		p.Pro278Ser	c.832C>T	Loss	**17.95**
CCNE1		copy number mean (9.81)		Gain	**5%:8.32, 95%:11.57**
PPP2R1A		p.Ser256Phe	c.767C>T	Gain	**26.22**
PIK3CA	**VH_1114**	p.Asn345Ile	c.1034A>T	Gain	**30.75**
PTEN		p.Leu139fs	c.416delT	Loss	**55.39**
FGFR2		p.Asn549Lys	c.1647T>A	Loss	**59.23**
MAX		p.His28Arg	c.83A>G	Gain	**26.81**
SMARCA4		p.Arg973Trp	c.2917C>T	Loss	**6.1**
PIK3CA	**VH_1115**	copy number mean (5.7)		Gain	**5%:4.81, 95%:6.75**
PIK3R1	**VH_1147**	p.Gln705Ter	c.2113C>T	Loss	**26.05**
MET		p.Glu168Asp	c.504G>T	Gain	**49.68**
PTEN		p.His123Tyr	c.367C>T	Loss	**29.86**
PTEN		p.Gln149fs	c.445_446insT	Loss	**30.47**
TP53		p.Gly244Cys	c.730G>T	Loss	**5.0**

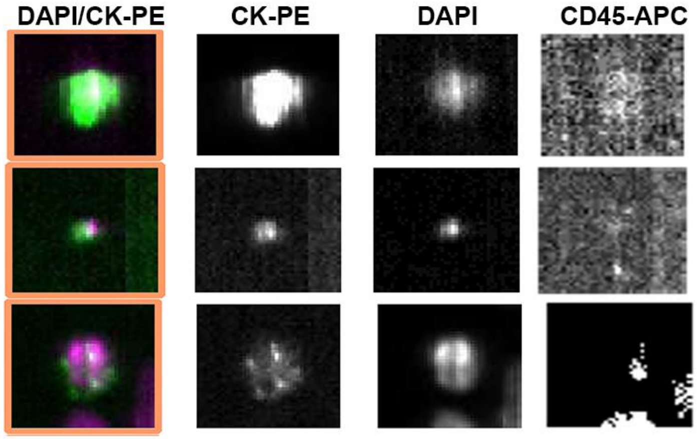

Figure S1. Representative examples of CTCs found in patients with EC (n = 36) using the CellSearch technology. Round-oval, DAPI^{+}, CD45^{-}, and CK^{+} cells were considered CTCs.

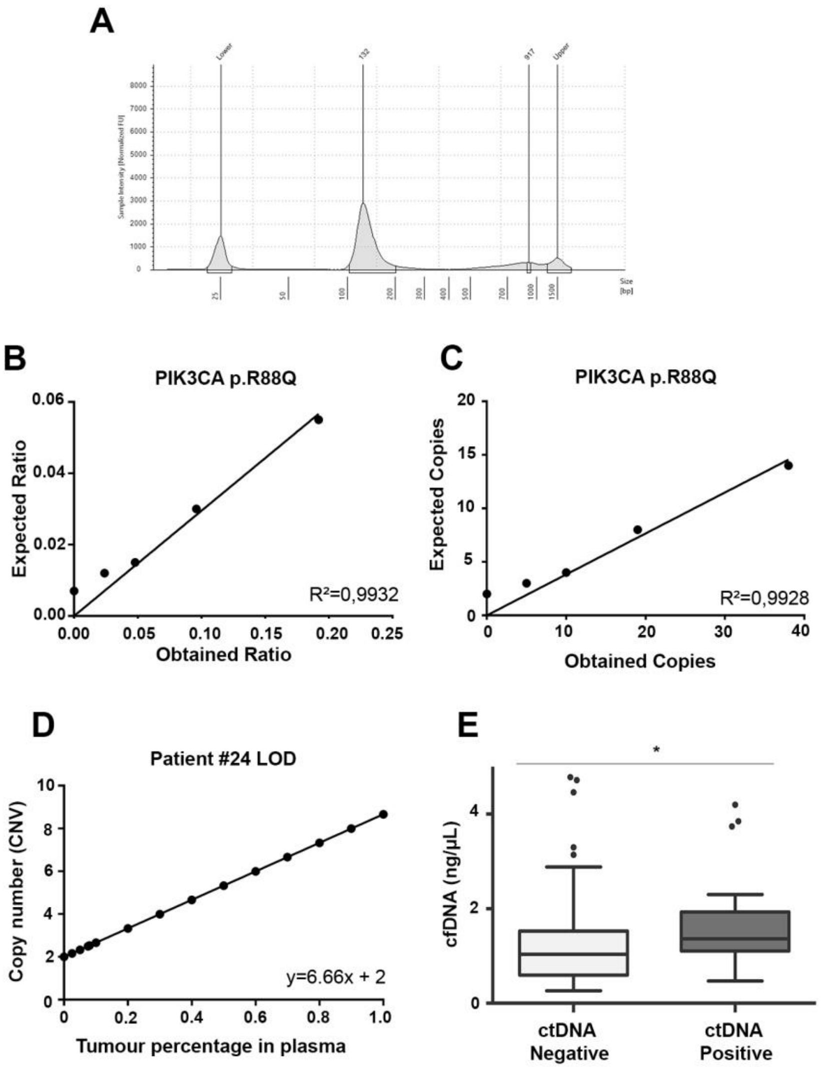
A
B
PIK3CA p.R88Q
Expected Ratio
Obtained Ratio
R²=0,9932
C
PIK3CA p.R88Q
Expected Copies
Obtained Copies
R²=0,9928
D
Patient #24 LOD
Copy number (CNV)
Tumour percentage in plasma
y=6.66x + 2
E
*
cfDNA (ng/µL)
ctDNA Negative
ctDNA Positive

Figure S2. (**A**) Fragment distribution of the cfDNA extracted from the plasma of patients with EC analyzed with TapeStationHS1000 (Agilent). (**B**) Representative example of the correlation between the expected and obtained ratios in a LOD ddPCR assay to detect a point mutation in *PIK3CA* (R88Q), LOD = 0.03%. (**C**) Correlation between the expected number of mutated copies and that obtained after ddPCR to detect a point mutation in *PIK3CA* (R88Q). (**D**) Representative example of the LOD to detect CNV of the *PIK3CA* gene, LOD = 8.00%.

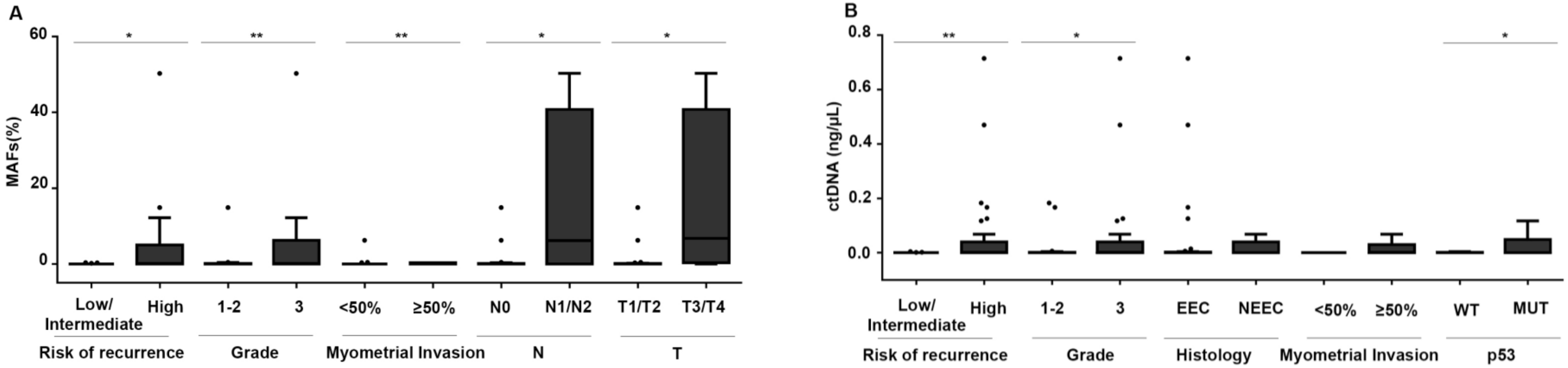

Figure S3. (**A**) ctDNA levels (MAFs) detected in EEC and grouped according to tumor grade, myometrial infiltration, risk of recurrence, and p53 status (n = 34). (**B**) ctDNA levels represented as ng/μL in EC and grouped according to tumor grade, myometrial infiltration, risk of recurrence, and p53 status (n = 51).

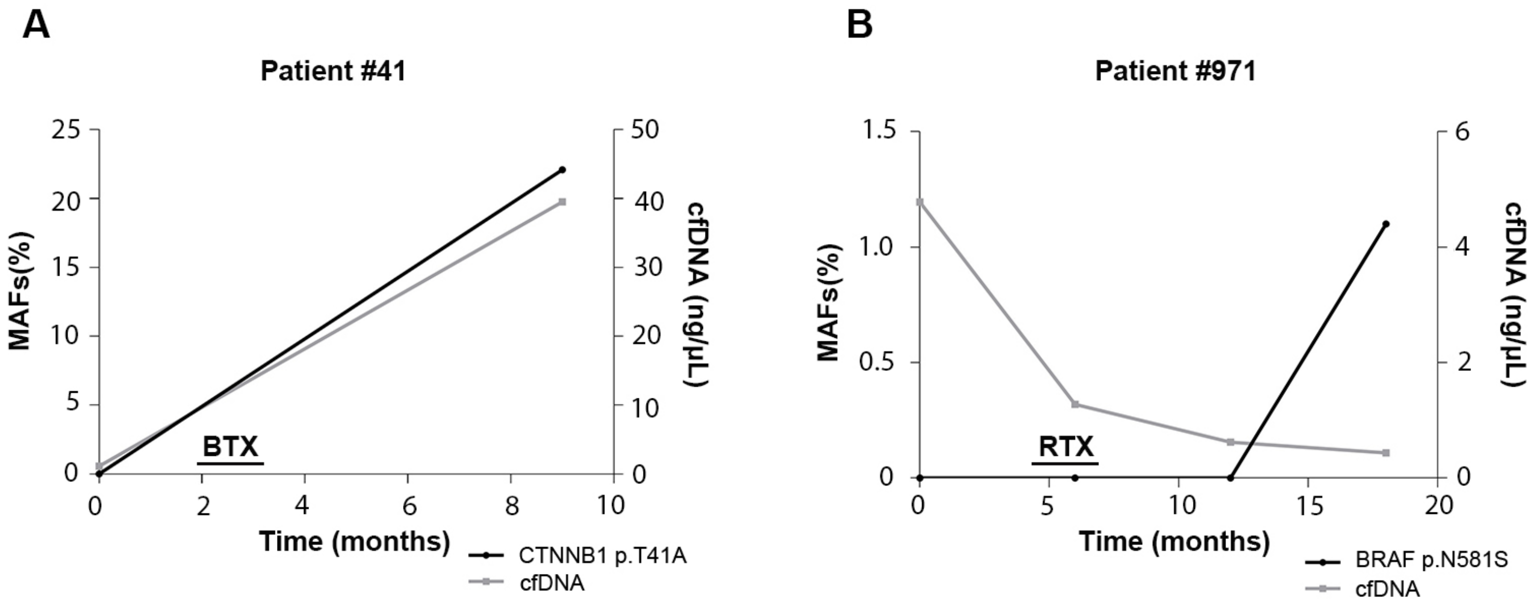

Figure S4. cfDNA and ctDNA levels during disease evolution in two patients with EC that progressed after surgery. (**A**) Patient #41 (EEC) progressed 7 months after surgery. A point mutation in *CTNNB1* (p.T41A) was found in the UA and then monitored at surgery and progression (PD) with ddPCR. (**B**) Patient #971 (EEC) progressed 18 months after surgery. A point mutation in *BRAF* (p. N581S) was found in the UA and then monitored at surgery and progression (PD) with ddPCR. RTX: radiotherapy; BTX: brachytherapy.

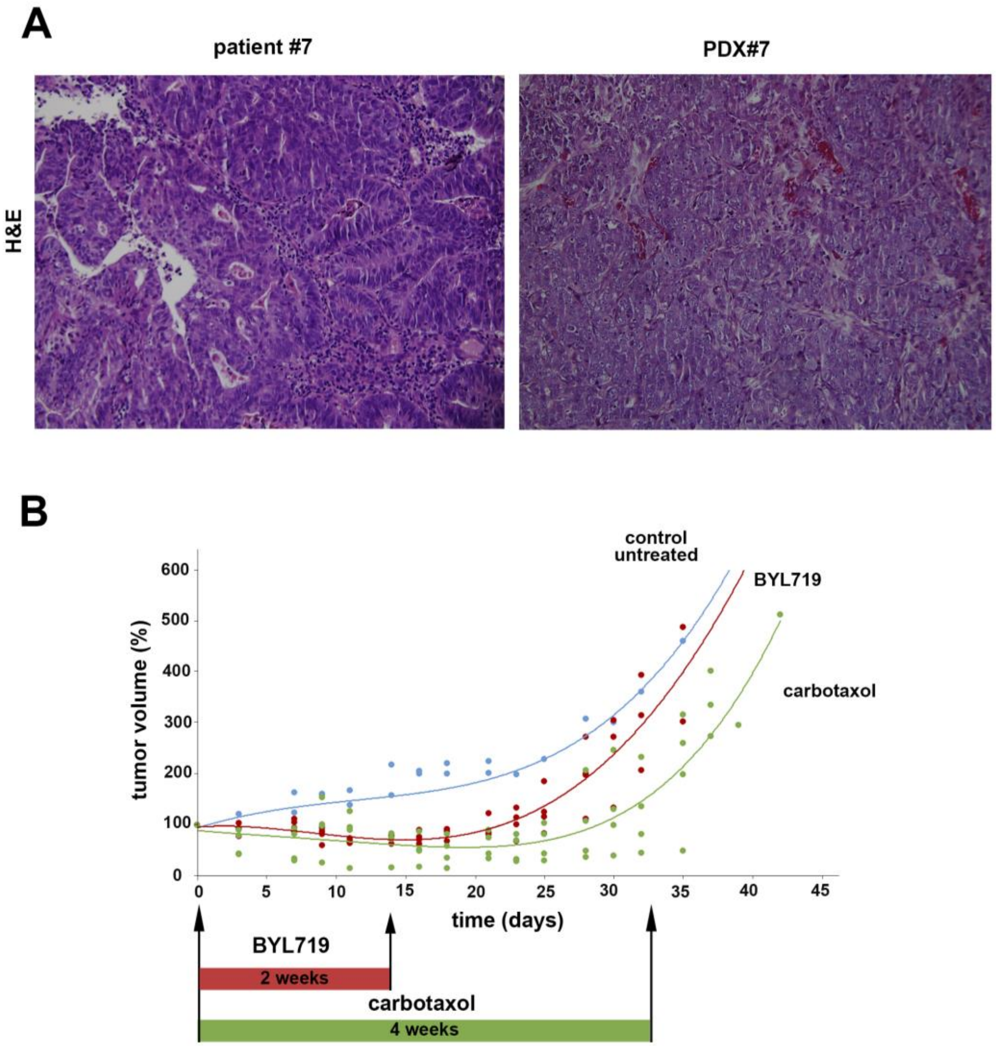

A
patient #7
PDX#7
H&E
B
control
untreated
BYL719
carbotaxol
tumor volume (%)
600
500
400
300
200
100
0
0
5
10
15
20
25
30
35
40
45
time (days)
BYL719
2 weeks
carbotaxol
4 weeks

Figure S5. PDX generation from UA as a preclinical model to test targeted therapies in Patient #7. (**A**) Hematoxylin and eosin staining showing the same type of histology in the primary tumor and the PDX. (**B**) PDX tumor growth evolution in response to the treatments. Twelve mice were distributed in three groups: a control untreated group, a control group with standard therapy of carboplatin/paclitaxel (weekly intraperitoneal injection for 4 weeks), and a group with BYL719 orally administered daily for 2 weeks, according to the literature. In the PDX #24 model, continuous administration of BYL719 was maintained until sacrifice because of the promising results with the BYL719 treatment (Figure 5).
